# Supplementary material for: Bio-Based Polyurethane Foams from Kraft Lignin with Improved Fire Resistance
Source: Polymers (Basel). 2023 Feb 21;15(5):1074. doi: 10.3390/polym15051074 (PMC10005662; doi:10.3390/polym15051074)
Supplement: Supplementary file 1 [file polymers-15-01074-s001.zip › Supplementary information.pdf]

Supplementary information

Bio-based polyurethane foams from kraft lignin with improved fire resistance

Fernanda R. Vieira [1,], Nuno V. Gama [1], Dmitry V. Evtuguin[1], Vitor Amaral [2] and Paula C. O. R. Pinto [3], Ana Barros-Timmons [1,*]*

*Corresponding author: fernanda.rvieira@ua.pt and anabarros@ua.pt

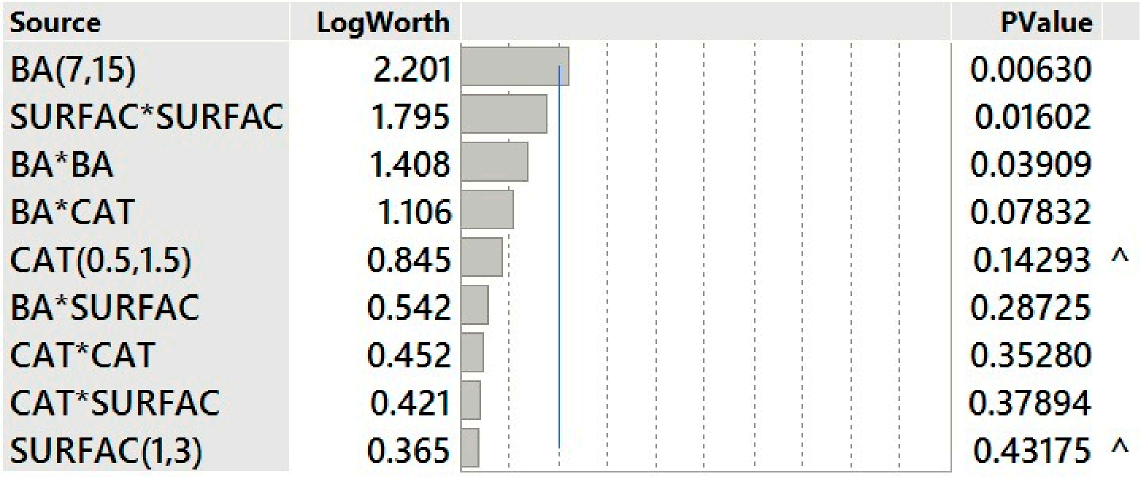

Figure S1. Pareto chart of the main effects obtained from the central composite design.

Table S1. ANOVA results for the empirical regression models for thermal conductivity and density.

Source	Responses					
	Thermal conductivity			Density		
	DF	SS	MS	DF	SS	MS
Model	9	5.44×10^{-5}	6.046×10^{-6}	9	128.22	14.247
Error	6	1.35×10^{-5}	2.253×10^{-6}	6	30.942	5.157
Total	15	6.79×10^{-5}	-	15	159.16	
F ratio	2.668			2.762		
p-value	0.1211			0.114		
R^2	0.800			0.805		
R^2 adjusted	0.5024			0.513		
Mean of response	0.0304			29.30		

DF: degree of freedom; SS: sum of square; MS: mean of square

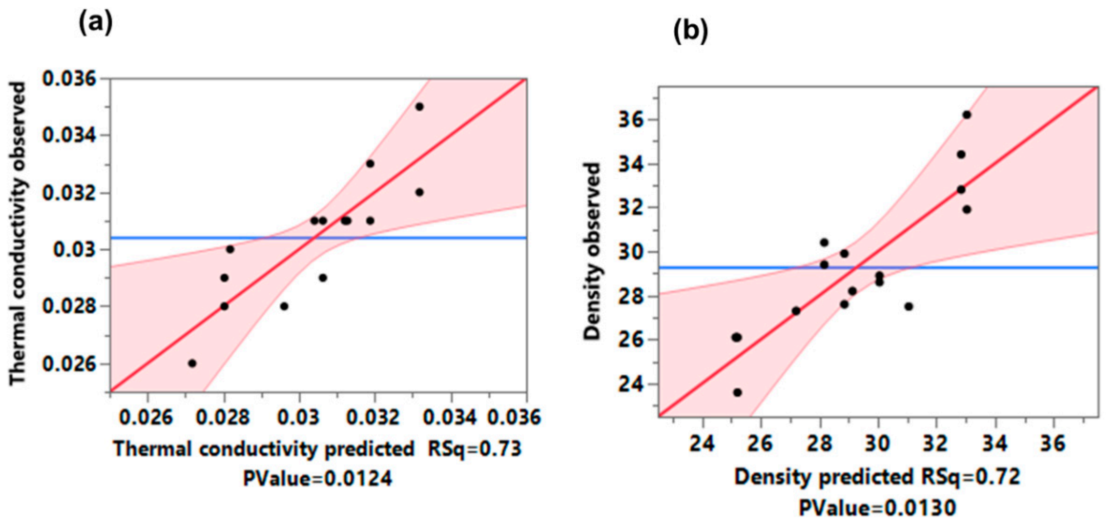

Figure S2. **(a)** Plots of observed versus predicted values of thermal conductivity, **(b)** Plots of observed versus predicted values of density from reduced model.

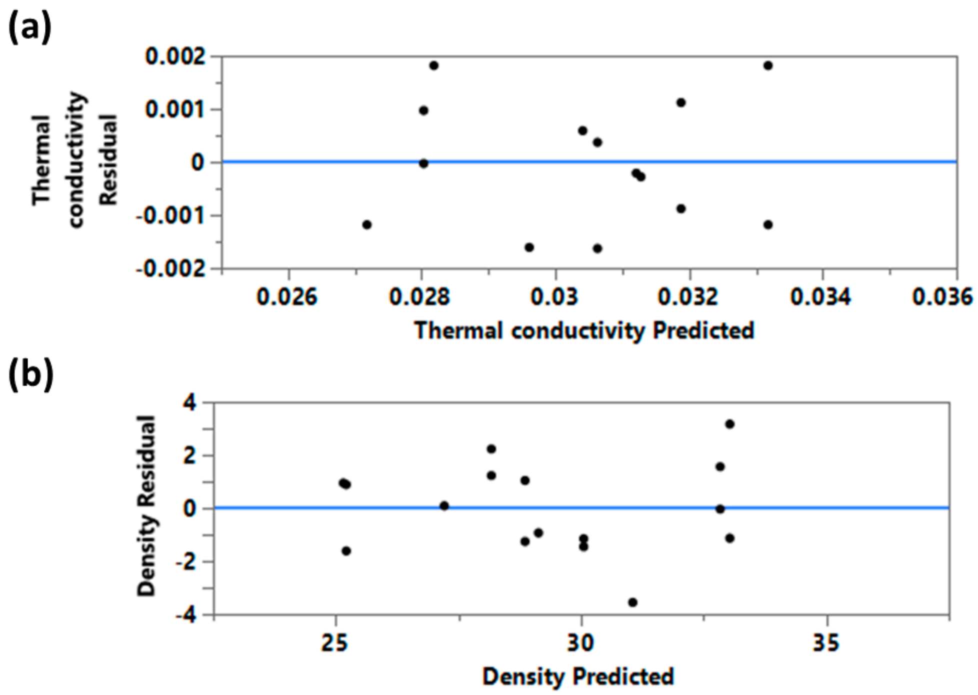

Figure S3. Plots of residual *versus* predicted values for **(a)** thermal conductivity and, **(b)** density obtained from reduced model.

(a)

Source	Nparm	DF	Sum of Squares	F Ratio	Prob > F
BA(7,15)	1	1	0.00000250	1.2834	0.2865
CAT(0.5,1.5)	1	1	0.00000640	3.2855	0.1033
BA*CAT	1	1	0.00001012	5.1977	0.0486*
BA*SURFAC	1	1	0.00000112	0.5775	0.4667
BA*BA	1	1	0.00002182	11.2004	0.0086*
SURFAC*SURFAC	1	1	0.00002255	11.5769	0.0078*

(b)

Source	Nparm	DF	Sum of Squares	F Ratio	Prob > F
BA(7,15)	1	1	87.025000	21.1124	0.0013*
CAT(0.5,1.5)	1	1	9.216000	2.2358	0.1691
BA*CAT	1	1	5.951250	1.4438	0.2602
BA*SURFAC	1	1	7.031250	1.7058	0.2239
BA*BA	1	1	0.013636	0.0033	0.9554
SURFAC*SURFAC	1	1	10.387636	2.5201	0.1469

Figure S4. (a) Effect test for thermal conductivity, **(b)** Effect test for density**.** * p-values are colored red if they are between 0.01 and 0.05, and p-values are colored orange if they are less than 0.01.
